# Management and effects of residual limbus inversion following closed reduction in developmental hip dysplasia: Protocol for a multicenter randomized controlled trial

**DOI:** 10.3389/fped.2022.1072831

**Published:** 2023-01-10

**Authors:** Chenyang Li, Weizheng Zhou, Yufan Chen, Federico Canavese, Lianyong Li

**Affiliations:** ^1^ Department of Pediatric Orthopedics, Shengjing Hospital of China Medical University, Shenyang, China; ^2^ Department of Pediatric Orthopedic Surgery, Lille University Centre, Jeanne de Flandre Hospital, Lille, France

**Keywords:** developmental dysplasia of the hip, residual acetabular dysplasia, closed reduction, residual limbus inversion, osteoarthritis, pediatric

## Abstract

Closed reduction is a common treatment method for developmental dysplasia of the hip (DDH) in children aged 6–18 months. Residual acetabular dysplasia (RAD) is the most common complication associated with closed reduction. Residual limbus inversion (RLI) is a common condition following DDH closed reduction. Previously, we confirmed that when limbus inversion exceeds 32.2% of the acetabular depth after closed reduction, RLI persists and leads to RAD; however, this was based on a small cohort with a short-term follow-up period. The long-term fate of RLI and the correlation between RLI and RAD have yet to be verified. Therefore, this multicenter clinical study protocol was designed in three parts to investigate the effect of RLI on acetabular development after closed reduction of DDH (a multicenter retrospective cohort study), effect of RLI clearance on acetabular development (a multicenter retrospective and prospective randomized controlled study), and influence of inverted limbus clearance on acetabular development during DDH reduction (a multicenter prospective cohort study). Statistical analysis was performed by assessing the basic measures of acetabular development including the acetabular index and central-edge angle using frontal pelvic radiographs; the magnitude of limbus inversion, cartilaginous acetabular index, and T1ρ mapping values were measured using magnetic resonance imaging. The multicenter retrospective cohort studies required 5 years of follow-up period at minimum, and the prospective randomized controlled studies required reviews of frontal pelvic radiographs every 6 months as well as data pooling every 2 years to compare the short- and mid-term outcomes of hip joint morphological development between the two groups of pediatric patients. This research program is expected to verify that RLI following closed reduction of DDH can affect acetabular development and that limbus excision during DDH reduction can improve postoperative RAD. Therefore, the indication and timing of surgical intervention for RLI after closed reduction of DDH provide a basis for revising the acceptable criteria for utilizing closed reduction of DDH to reduce the incidence of osteoarthritis caused by RAD following DDH treatment.

**Clinical Trial:** http://www.chictr.org.cn/showproj.aspx?proj=35045 (ChiCTR1900020996)

## 1. Introduction

Closed reduction is a common method for treating developmental dysplasia of the hip (DDH) in children aged 6–8 months; however, re-dislocation after reduction, avascular necrosis, residual acetabular dysplasia (RAD), and other complications cannot be completely avoided (1). Among these, RAD is the most common complication of DDH closed reduction treatment. A prospective multicenter study conducted by Sankar et al. (2) found that even with stable closed reduction, approximately 11% of children still had residual dysplasia, and long-term follow-up results of a study by Terjesen (3) noted a higher statistic of up to 38%. RAD is the main cause of osteoarthritis (OA) in young adults (4, 5) which often requires total hip replacement (THR). Approximately 9.1% of THR is caused by RAD, and among young adults, this proportion is as high as 21%–29% (6, 7). Therefore, prevention of RAD after DDH treatment and timely correction of RAD are key to long-term prevention of OA.

The causes of RAD in children after closed reduction for DDH are complex. Age, dislocation height, and acetabular index at reduction are commonly used clinical indicators to predict RAD (3, 8, 9), but these factors can only partially explain the causes of RAD from clinical phenomena. Several factors affect the development of the acetabulum after closed reduction. In addition to the genetic quality of DDH, the quality and development potential of the acetabular cartilage, the soft tissue in the acetabulum, and the evolution of the concentric relationship between the acetabulum and femoral head are related to the shaping ability of the hip joint. A relevant understanding of the internal causes of RAD and timely intervention are of clinical significance for the prevention of OA caused by RAD. Previous clinical studies based on indirect radiological observations cannot directly reflect real world practice, in which the non-bone structure and concentric evolution of the hip joint must be considered. Radiological observations can be used to monitor acetabular development; however, they cannot accurately predict RAD (10, 11). Therefore, a method for follow-up observation of hip joint development is warranted to enable better clinical understanding of RAD.

It is generally accepted that the labrum is a tough fibrocartilaginous structure attached to the rim of the osseous acetabulum. Before skeletal maturity, it is characterized by non-ossifying soft tissue which helps to stabilize the femoral head into the acetabulum until osseous acetabular rim merge (12). Limbus inversion remains a common phenomenon after closed reduction, which increases the gap between acetabular and femoral head combined with ligamentum teres, and reduces the quality of closed reduction regarding to concentricity. That's why clearance of interposition (inverted limbus and ligamentum teres) is beneficial to sustain the concentricity and acetabular development. But the everted part of limbus was reserved to keep the potentiality to osseous acetabular rim. It's our negligence that ambiguous expression makes you confused about the management. we have made correction into our manuscript as follow. Clearance of interposition is performed by a standard anterior and superior approach to the hip, and remove ligamentum teres, the transverse ligament of the acetabulum and inverted limbus without the everted portion.

**Figure 1:**
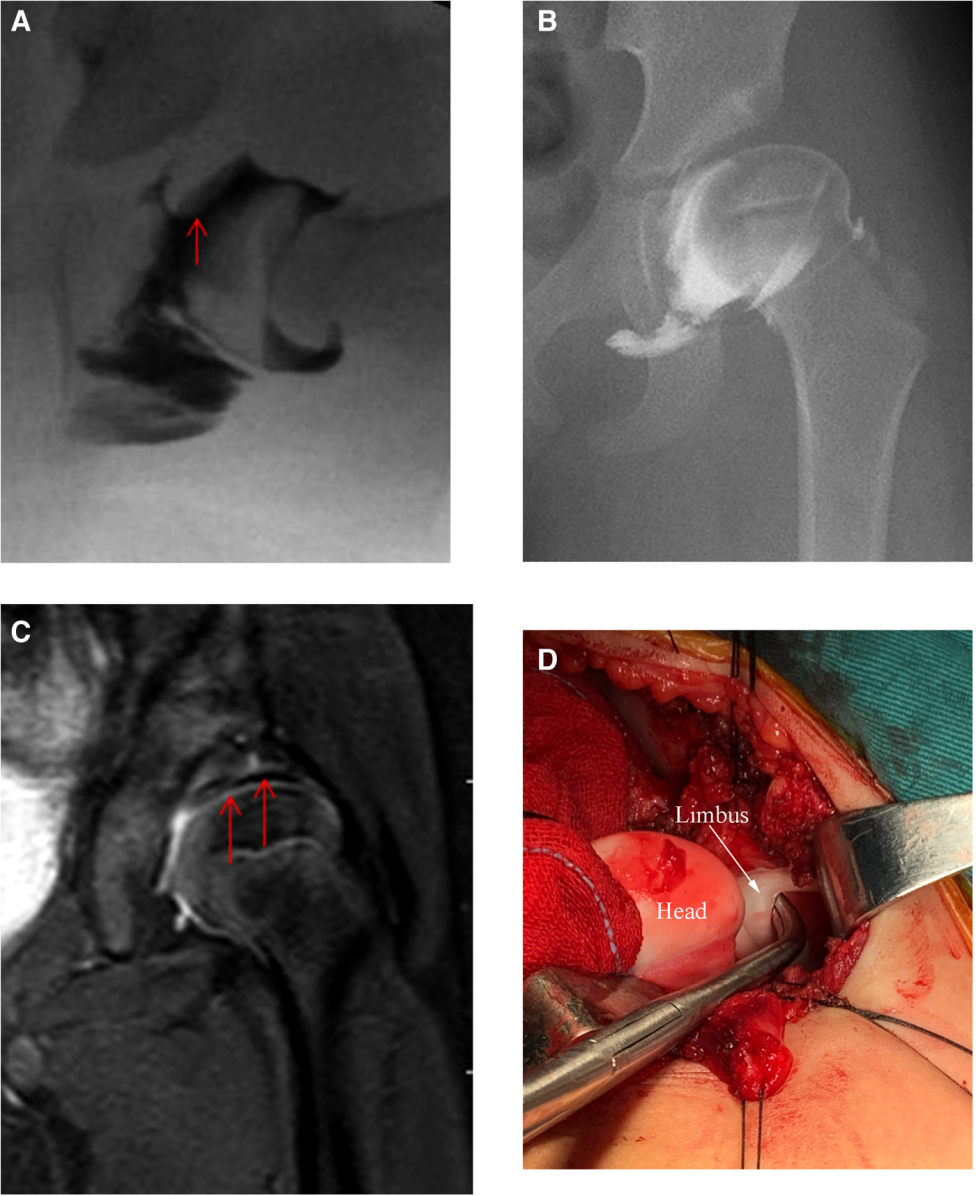
A 14-month-old female patient who underwent closed reduction of left hip dislocation. (**A**) Arthrography showed hypertrophic inverted limbus tissue during reduction; (**B**) Residual acetabular dysplasia after reduction for 3 years. Arthrography showed no abnormal tissue between the femoral head and acetabulum; (**C**) Coronal MRI showed a thin layer of hypointense tissue embedded in the superior joint space (arrows); (**D**) Intraoperatively confirmed limbus inversion.

Magnetic resonance imaging (MRI) can display all bone and non-bone structures of the hip joint from multiple planes; therefore, it is useful for observing development of the acetabulum after closed reduction of DDH. However, MRI is commonly used to evaluate the pathological morphology of DDH before reduction and is seldom used for long-term follow-up (13, 14). In 2020, we published prospective MRI long-term follow-up results after closed reduction of DDH and established that “safety” (a reduction which could be maintained by the abduction of <65°) and “stability” (Ramsey safe zone >30°) are acceptable closed reduction standards (15). Owing to shaping of the pathological structure of soft tissue after reduction, non-concentric reduction of the DDH can gradually achieve concentric reduction. Additionally, we found an interesting phenomenon during follow-up using serial MRI: the inverted limbus was not absorbed overtime; instead, it was compressed into a thin layer of fibrous tissue inter-positioning in the superior joint space (15). Previous radiographic studies have shown that the inverted limbus can be absorbed and can disappear with gradual “docking” of the femoral head after reduction, but this is not the case. Intraoperative arthrography has shown that the inverted limbus is a hypertrophic filling defect with a widening superior joint space (Figure 1A); although this cannot be observed during the course of medium-term follow-ups (Figure 1B), MRI can reveal the residual inverted limbus as a thin layer of hypointense tissue embedded in the superior joint space (Figure 1C), as confirmed during surgery correcting for RAD (Figure 1D). This phenomenon has not been reported previously.

We conducted observational MRI follow-up for an average of 50 months in 26 children (29 hips) with an inverted limbus immediately after closed reduction. At the last follow-up, 76% of the patients maintained residual limbus inversion (RLI). ROC curve analysis showed that when the degree of limbus inversion exceeded 32.2% of the acetabular depth after closed reduction, RLI persisted. Furthermore, we also observed that development of the acetabular index of RLI was significantly lesser than that of the inversion absorbed group. This suggested that RLI leads to delayed ossification of the acetabular cartilage, which in turn leads to RAD. This finding provides a new perspective and basis for renewed understanding of RAD in clinical practice (16). Although we established the relationship between RLI and RAD, our findings were based on a small sample cohort that did not include follow-up until skeletal maturity. Therefore, design of a multicenter randomized controlled trial is warranted to elucidate the correlation between RLI and RAD and whether RLI affects the development of other hip joint components. We performed retrospective and prospective multicenter randomized controlled studies to address these questions.

## 2. Methods and analysis

### 2.1. Objectives

Limbus inversion is a common pathological change in DDH; however, how does the RLI evolve after closed reduction, and how does it affect development of the acetabulum? To date, there have been no reports addressing these questions. Whether RLI affects the development of other aspects of the hip joint, whether surgical intervention is required, and whether acetabular dysplasia can be reversed after intervention are practical issues that must be addressed urgently. In light of the limitations of our previous study, we aim to address these issues through a retrospective and prospective multicenter randomized controlled study. The main purpose of this study is to clarify the long-term outcome of RLI after DDH closed reduction and its impact on development of the acetabulum. We aim to determine the appropriate indication and timing of surgical intervention for RLI after DDH closed reduction and to provide a basis for revising the acceptable criteria for closed reduction of DDH, to reduce the incidence of OA caused by RAD after DDH treatment.

### 2.2. Study design

#### 2.2.1. Long-term effects of RLI on acetabular development after closed reduction of DDH: a multicenter retrospective cohort study

**Figure 2:**
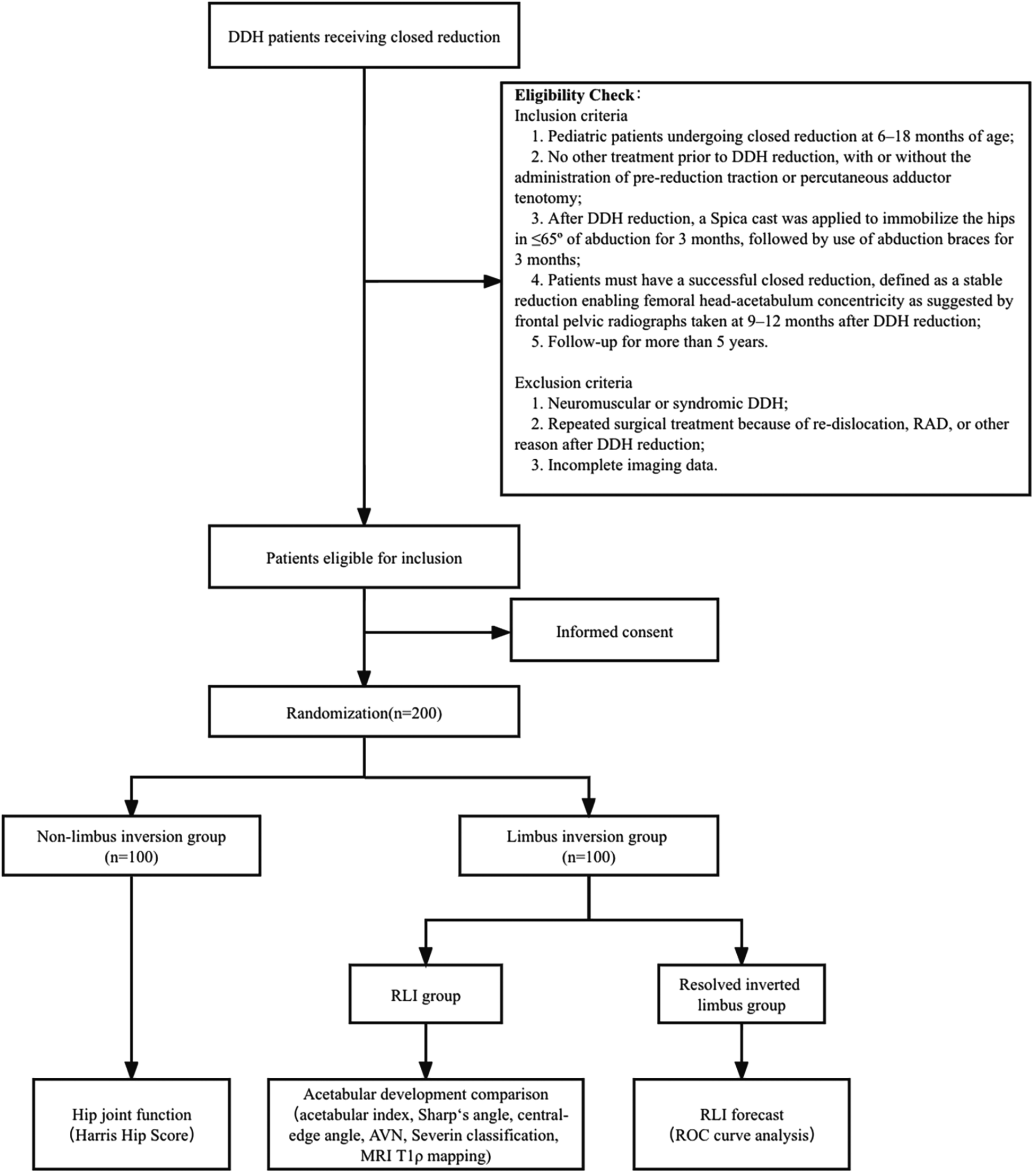
Flow diagram of the multicenter retrospective cohort study.

We retrospectively analyzed the outcomes of RLI as well as the mid- and long-term impact of RLI on acetabular development in DDH patients receiving closed reduction at different centers. The trail is illustrated in a flowchart as shown in Figure 2.

##### 2.2.1.1. Inclusion criteria

1. Pediatric patients undergoing closed reduction at 6–18 months of age;
2. No other treatment prior to DDH reduction, with or without the administration of pre-reduction traction or percutaneous adductor tenotomy;
3. After DDH reduction, a Spica cast was applied to immobilize the hips in ≤65° of abduction for 3 months, followed by use of abduction braces for 3 months;
4. Patients must have a successful closed reduction, defined as a stable reduction enabling femoral head-acetabulum concentricity as suggested by frontal pelvic radiographs taken at 9–12 months after DDH reduction;
5. Follow-up for more than 5 years.

##### 2.2.1.2. Exclusion criteria

1. Neuromuscular or syndromic DDH;
2. Repeated surgical treatment because of re-dislocation, RAD, or other reason after DDH reduction;
3. Incomplete imaging data.

##### 2.2.1.3. Cohort follow-up

**Figure 3:**
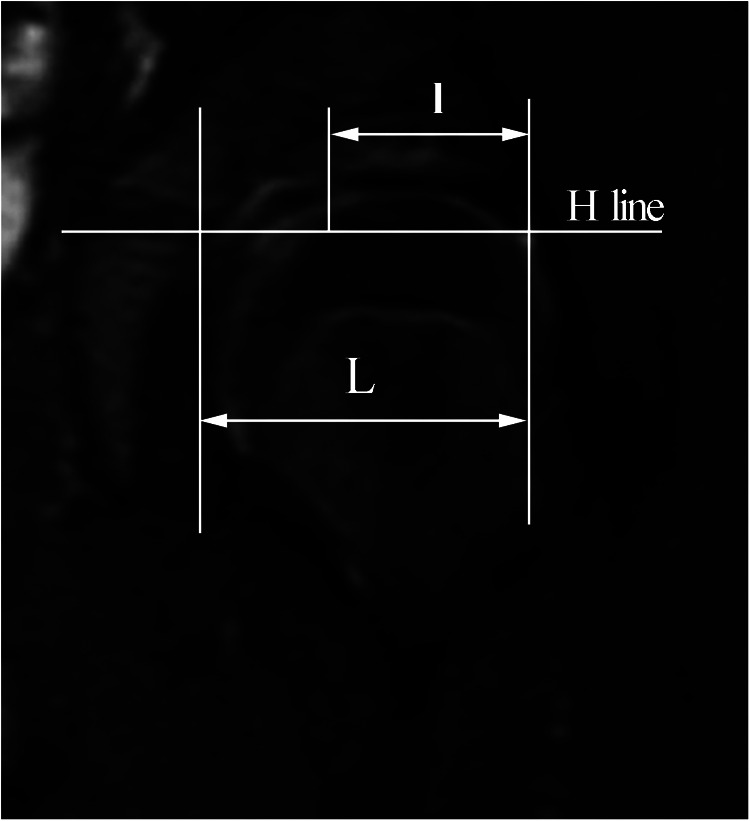
Schematic diagram of MRI limbus inversion degree measurement. “*l*” is the horizontal depth of the limbus insertion, and “*L*” is the horizontal depth of the ilium-acetabular roof. The degree of limbus inversion is *t* = *l/L *× 100%.

The frontal pelvic radiographs in a standing position and MRI images of the hip joints with the lower limbs in a neutral position were taken during follow-up, and the function examination and measurements were as:

1. Visual analogue scale (VAS) and Harris hip score (HHS) was used to evaluate hip joint function. As preschool children cannot describe pain precisely, we decide to change our measurements according to your recommendation. For preschoolers who are unable to describe their specific situation, along with Harris hip score, a visual analogue scale (VAS) was also applied for the assessment of pain severity of participants (17). In order to determine VAS score, a 100-mm horizontal line without scaling was designed in which 0 was marked as “no pain in hip” and 10 was marked as “unbearable pain”. Participants were then instructed to place a vertical mark reflecting their soreness severity.
2. The acetabular index, Sharp's angle, cartilaginous acetabular index, and central-edge angle were measured using frontal pelvic radiographs; the morphology of the hip joints was evaluated using the Severin classification.
3. The presence of RLI was observed on T2-weighted fat-suppressed MRI sequences of the hip joints. If RLI was present, the magnitude of limbus inversion was measured at level with the center of the triangular cartilage (16) (Figure 3, schematic diagram of MRI limbus inversion degree measurement).
4. MRI T1ρ mapping.

The application of MRI T1ρ mapping to assess the quality of the acetabular cartilage has been a research hotspot in recent years. MRI T1ρ mapping can quantitatively evaluate the proteoglycan content of articular cartilage and is a sensitive indicator for measuring proteoglycan content. The T1ρ mapping values are inversely proportional to proteoglycan content. The T1ρ mapping values of the anterior, superior, and posterior acetabular cartilage were measured on the sagittal planes passing through the medial third and the lateral third of the acetabular roof, respectively.

**Figure 4:**
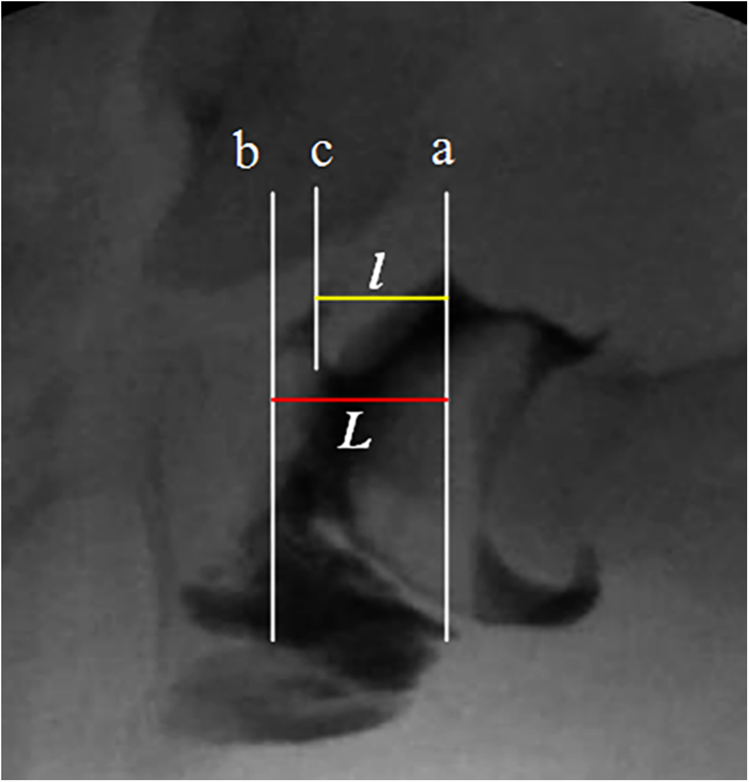
Schematic diagram of measuring the degree of limbus inversion in arthrography. Line a is the vertical line passing through the limbus and the folded joint capsule, line b is the vertical line that fills the innermost edge of the joint space (the floor of the acetabulum), and line c is the vertical line that passes through the innermost edge of the varus limbus; “*l*” is the limbus embedded horizontal depth, “*L*” is the horizontal depth of the acetabulum. The degree of limbus inversion is *t* = l/L × 100%.

5. Avascular necrosis (AVN) of the femoral head was assessed based on the Kalamchi–MacEwen criteria.
6. The initial magnitude of limbus inversion was measured using arthrography taken at closed reduction (Figure 4, same as the measurements on MRI).

Each parameter is measured by 2 raters in respective institution. Furthermore, the reliability and consistency are also conducted.

##### 2.2.1.4. Statistical analysis

Statistical analysis was performed using SPSS 27.0 software (IBM, SPSS Inc.).

1. To ensure consistency and reliability between multicenter research samples, it is first necessary to conduct a consistency analysis on the structural data, such as age and sex, of the included cases from each center and the International Hip Dysplasia Institute (IHDI) classification using the Cohen's kappa statistic. Simultaneously, the baseline characteristics (such as structural composition and pathological severity) of the included cohorts within each center must be consistent; imbalances in baseline characteristics were corrected by increasing the sample size.
2. The included patients were divided into the non-limbus inversion group (A) and the limbus inversion group based on the presence of limbus inversion at closed reduction. The limbus inversion group was then further divided into the RLI group (B) and the resolved inverted limbus group (C) according to the state at final follow-up. A combination consisting of any two of the groups was organized into cohorts. Fisher's precision probability test was used to compare the difference between the sexes and affected sides, using analysis of variance to compare the different ages between the two groups. Differences in acetabular index was compared using Student's *t*-test. Chi-square test was used to compare IHDI classification, VAS and HHS, and Severin classification at the final follow-up, and analysis of variance was used to compare the MRI T1ρ mapping values and the difference in the initial magnitude of limbus inversion between groups B and C, as well as the difference between the initial magnitude of limbus inversion and that at the final follow-up in group B.
3. ROC curve analysis was used to predict the cut-off value of the initial magnitude of limbus inversion in the case of persistent RLI as well as calculate its specificity, sensitivity, and statistical significance.
4. Sample size calculation.
  In this study, ossification of the acetabular cartilage was the main observation; thus, improvement of the acetabular index was the most suitable observation indicator. The sample size calculation was based on the previously published acetabular index at the final follow-up in the group with RLI and the group without RLI after closed reduction (21.41°±3.61° and 18.88 ° ± 4.32° in the two groups, respectively) (15). The significance level *α*, the statistical power of the test, the ratio of the sample sizes in the two groups, and the percentage of patients lost to follow-up were set at 0.05, 0.9, 1:1, and 20%, respectively. The minimum sample size for each group was calculated to be 67 cases (134 cases in total). Therefore, at least 67 cases needed to be enrolled in the observation group and the control group, respectively.

#### 2.2.2. Effects of RLI clearance on acetabular development: a retrospective and prospective multicenter randomized controlled study

##### 2.2.2.1. Selection of participants for the retrospective study

**Figure 5:**
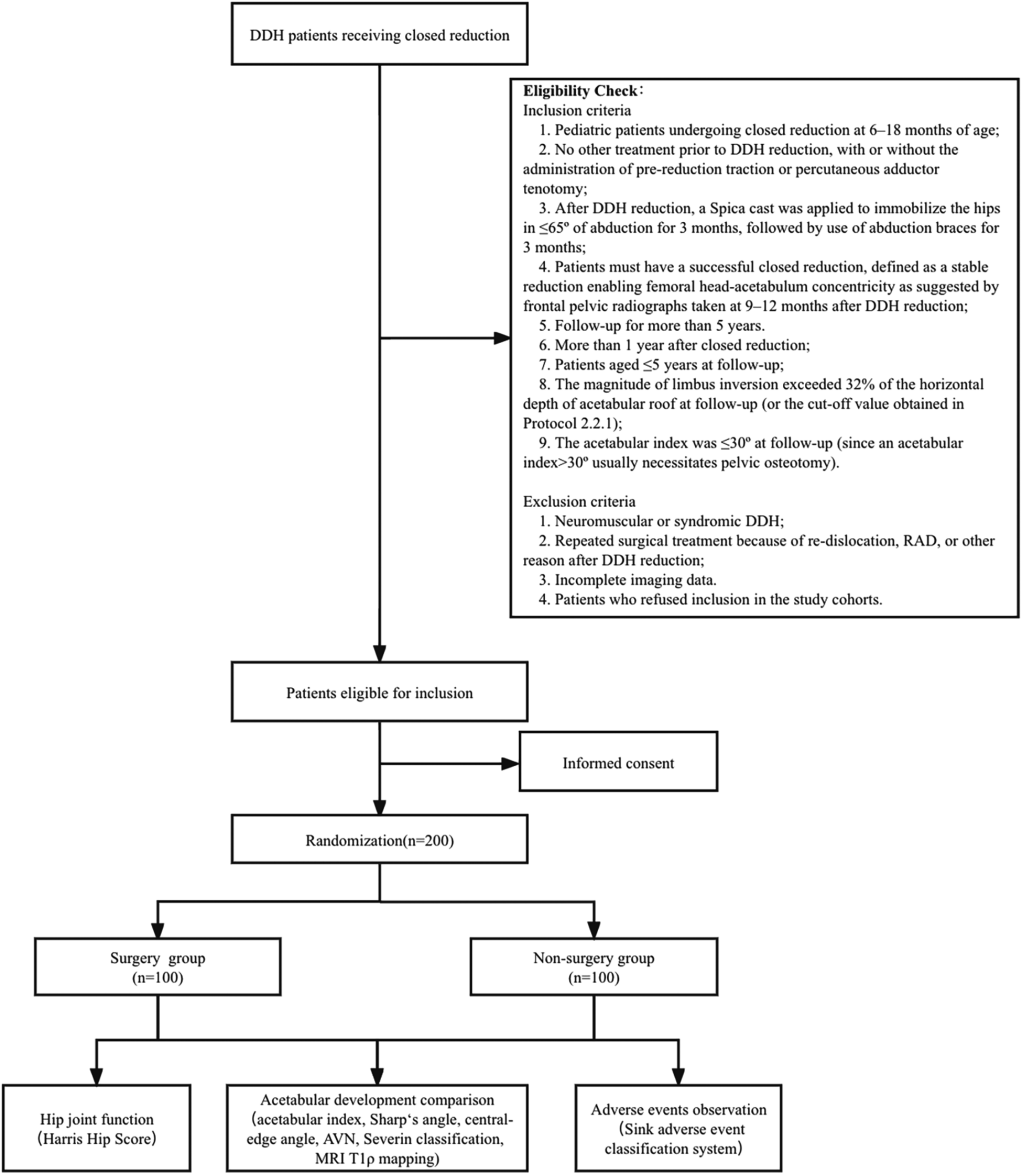
Flow diagram of the retrospective and prospective multicenter randomized controlled study.

To maximize the use of the peak ossification potential of acetabular cartilages in patients before the age of 5 years, pediatric patients who underwent successful closed reduction between 6 and 18 months that had experienced RLI and were ≤5 years old at follow-up were selected as study participants, given that it is more instrumental to observe the reversibility of delayed acetabular cartilage ossification. The frontal pelvic radiographs and MRI images of the hip joint were taken during follow-up to evaluate limbus inversion. The trail is illustrated in a flowchart as shown in Figure 5.

###### 2.2.2.1.1. Inclusion criteria

Inclusion criteria 1–5 are the same as the previously listed inclusion criteria 1–5 (2.2.1.1);

6. More than 1 year after closed reduction;
7. Patients aged ≤5 years at follow-up;
8. The magnitude of limbus inversion exceeded 32% of the horizontal depth of acetabular roof at follow-up (or the cut-off value obtained in Protocol 2.2.1);
9. The acetabular index was ≤30° at follow-up (since an acetabular index>30° usually necessitates pelvic osteotomy).

###### 2.2.2.1.2. Exclusion criteria

Exclusion criteria 1–3 are the same as previously listed exclusion criteria 1–3 (2.2.1.2);

4. Patients who refused inclusion in the study cohorts.

For pediatric patients satisfying the inclusion/exclusion criteria, the basic measures of acetabular development including the acetabular index and central-edge angle were assessed using frontal pelvic radiographs; the magnitude of limbus inversion, cartilaginous acetabular index, and T1ρ mapping values were measured using MRI.

##### 2.2.2.2. Randomized blocks and interventions

A randomized trial study design was the most appropriate approach to observe the efficacy of surgical intervention, with blinding techniques used during statistical analysis of the data. A double-blind study was not feasible because the surgeon was required to provide detailed information on the surgical regimen. The researchers in each center divided the RLI participants into two study cohorts based on the randomized block design, i.e., the surgery and non-surgery groups. After informed consent was obtained from the guardians of patients in the surgery group, clearance of the inverted limbus *via* arthrotomy was performed.

All surgeries were performed by pediatric hip joint surgeons with senior job titles *via* a bikini incision, and the inverted limbus tissue embedded between the acetabulum and femoral head was excised after arthrotomy without damaging the limbus-cartilage complex. After surgery, casts or braces were applied to immobilize single hips in abduction for 4 weeks and the patients gradually resumed walking after removal of external fixators. No special rehabilitation therapy was administered.

##### 2.2.2.3. Prospective follow-up observation

Prospective follow-up observation was performed for the two clinical cohorts, and frontal pelvic radiographs were reviewed every 6 months. Since development of the hip joint in children is a long-term process, the included pediatric patients needed to be followed until skeletal maturity. Data pooling was performed once every 2 years to compare the short-term and medium-term outcomes of hip joint morphological development between the two groups. MRI T1ρ mapping was used to quantitatively evaluate quality of the acetabular cartilage before and during postoperative follow-up. The evaluated aspects and parameters were the same as those specified in 2.2.1.

##### 2.2.2.4. Adverse events observation

Complications and adverse events in the surgical treatment group were recorded and classified according to the adverse event classification system described by Sink et al. (DOI:10.1007/s11999-012-2343-2). Grade I: no clinical relevance and no need to change routine postoperative care; Grade II: resolving spontaneously after simple outpatient management or outpatient observation; Grade III: requiring hospitalization or surgical treatment before resolution; Grade IV: chronic impairment of body functions. Grade III and IV events were included in the analysis of results.

##### 2.2.2.5. Bias control

To control bias as much as possible, blinding techniques were adopted for data measurement and analysis, i.e., after the imaging data from each center were summarized into research groups, the grouping of clinical data was blinded to the data measurers; at the same time, a consistency test was conducted on the basic situation of the cohort composition within each center (in the same manner as study protocol 2.2.1) to control bias caused by differences in sample composition at different centers.

##### 2.2.2.6. Statistical analysis

Fisher's precision probability test was used to compare the difference between the sexes and affected sides, using analysis of variance to compare the different ages between the surgical and non-surgical cohorts. Differences in acetabular index was compared using Student's *t*-test. Chi-square test was used to compare IHDI classification, VAS and HHS, and Severin classification at the final follow-up, and analysis of variance was used to compare the MRI T1ρ mapping values and the incidence of Grade III and Grade IV adverse events.

#### 2.2.3. Influence of inverted limbus clearance on acetabular development during DDH reduction: a multicenter prospective cohort study

##### 2.2.3.1. Research object

**Figure 6:**
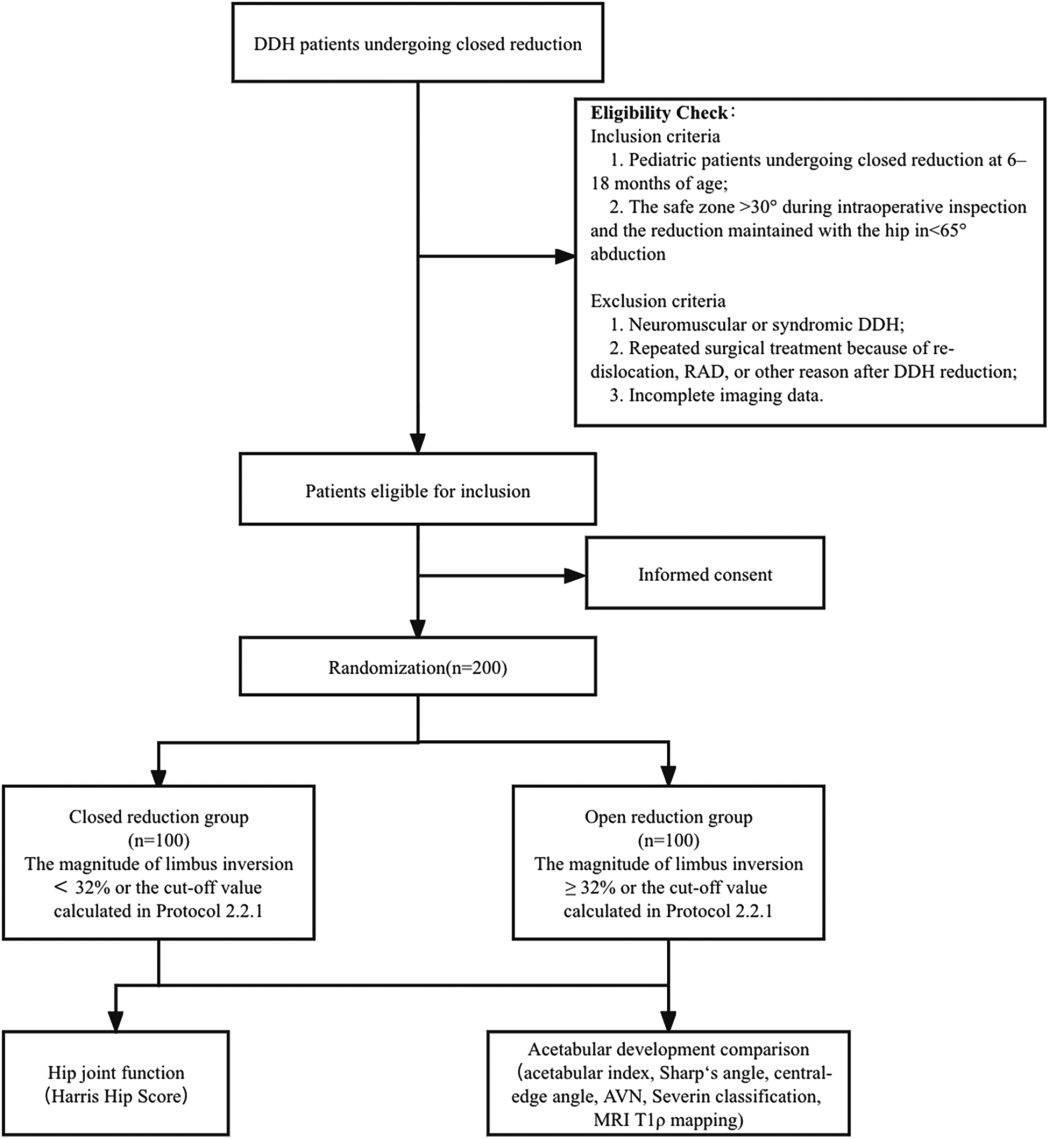
Flow diagram of the multicenter prospective cohort study.

In each center, the quality of reduction was first evaluated intraoperatively based on our published “safety and stability” criteria for an acceptable closed reduction (15), i.e., the safe zone >30° during intraoperative inspection and the reduction maintained with the hip in <65° abduction. DDH patients undergoing closed reduction at 6–18 months who satisfied the criteria were selected as participants for observation. The exclusion criteria are the same as those specified in study protocol 2.2.1. In the case of <70° abduction before DDH reduction, percutaneous adductor longus tenotomy was performed. The trail is illustrated in a flowchart as shown in Figure 6.

##### 2.2.3.2. Cohort grouping and interventions

For cases meeting the assessment criteria specified in 2.3.1, the labral morphology was further evaluated using intraoperative arthrograms if there was no limbus inversion or if magnitude of the limbus inversion was <32% of the horizontal depth of the acetabular roof (or the cut-off value obtained in 2.2.1). A closed reduction using Spica cast immobilization was then performed to construct the control cohort. If intraoperative evaluation suggested that the magnitude of limbus inversion exceeded the cut-off, open reduction with post-reduction Spica cast immobilization was performed, in which the acetabular soft tissues were removed *via* the bikini incision approach, and the inverted limbus was excised. Patients undergoing open reduction procedures constituted the experimental cohort. Patients in the two cohorts underwent post-reduction cast immobilization for 3 months followed by immobilization with braces for 3 months.

##### 2.2.3.3. Prospective follow-up observation

The control and experimental cohorts were observed radiologically every 6 months during follow-up, and the short-, mid-, and long-term outcomes of hip joint morphological development were compared between the two groups of pediatric patients. Data pooling was performed once every 2 years, and clinical function tests, radiologic measurements, and MRI T1ρ mapping measurements were performed at the stage of skeletal maturity.

##### 2.2.3.4. Statistical analysis

Fisher's precision probability test was used to compare the difference between the sexes and affected sides, using analysis of variance to compare the different ages between the surgical and non-surgical cohorts. Differences in acetabular index was compared using Student's *t*-test. The chi-square test was used to compare IHDI classification, VAS and HHS, and Severin classification at the final follow-up, and analysis of variance was used to compare the MRI T1ρ mapping values, re-dislocation rate, incidence of RAD, and incidence of AVN between the two cohorts.

### 2.3. Outcome measures

#### 2.3.1. Primary outcome measures

1. The improvement of the acetabular index
2. Severin classification
3. The magnitude of limbus inversion
4. Avascular necrosis (AVN) of the femoral head was assessed based on the Kalamchi–MacEwen criteria.

#### 2.3.2. Secondary outcome measures

1. VAS and HHS
2. The T1ρ mapping values

## 3. Discussion

DDH is one of the most common limb deformities in the skeletal system of infants, and its treatment methods usually depend on the pediatric patient's age and the severity of hip joint dislocation and dysplasia. Patients younger than 6 months of age can be treated nonoperatively, whereas pediatric DDH patients aged >6 months or those experiencing failure in treatment with the Pavlik harness require surgical treatment. Closed reduction is an important surgical procedure for achieving concentric reduction of the joint; however, the incidence of post-reduction RAD remains high. The labrum is a fibro-chondral structure attached to the edge of the acetabulum (18). It surrounds the acetabulum in a horseshoe shape, and its ends are connected *via* the transverse ligament, which increases depth of the acetabulum cavity, expands coverage of the femoral head, and enhances joint stability (19). In contrast, the limbus in DDH becomes hyperplastic, hypertrophied, rounded, and inverted relative to the edge of the acetabulum owing to the loss of stimulus for normal growth and the remodeling effect exerted by the femoral head. In morphology, the labrum in DDH is completely different from those observed in normal hips; thus, the pathologic labrum in DDH was referred to as a “limbus” to distinguish it from the morphologically normal labrum. The limbus becomes hypertrophied and surrounded by fibrous tissues, which is a key barrier to concentric reduction (20). With the exacerbation of limbus inversion, stenosis at the entrance of the acetabular cavity becomes increasingly noticeable, which often leads to failure in closed reduction (21, 22). However, RLI is still common even after safe and stable closed reduction is achieved.

At present, the changes and outcomes of the inverted limbus after closed reduction of DDH remain controversial. Severin et al. (23) believed that after achieving stable closed reduction, the inverted limbus would be gradually absorbed and disappear; however, others believed that the inverted limbus was a key factor leading to AVN. Previous studies have been based on indirect observation of x-ray images, and there is a lack of direct evidence regarding the morphological evolution of the inverted limbus. This study used serial MRIs to observe the outcomes of limbus inversion after closed reduction of DDH, which lessened the impact of radiation on pediatric patients and enhanced the safety and reliability of the study. Our previous findings indicated that the magnitude of limbus inversion after closed reduction is closely related to its medium-term morphological outcome (16). However, the previous study had certain limitations since it was based on a small sample and did not follow-up until skeletal maturity. Previous studies have confirmed that a primary limbus inversion leads to primary acetabular dysplasia and early-onset OA (24, 25), but the long-term result of RLI has not been reported in DDH patients who previously underwent closed reduction.

Reaching skeletal maturity of the hip is a long-term process; therefore, the prospective design of this protocol better reflects the relationship between RLI and multiple prognostic outcomes while balancing many known or unknown biases. This research plan is to be led by the corresponding author's institution and carried out by multiple clinical research centers in cooperation with pediatric orthopedic centers in China. Compared to previous case-control studies, this study was designed as a multicenter clinical study with a higher level of evidence. Consistency of the characteristics included in a multicenter sample study is a key issue in clinical research. In the process of sample collection, cohort characteristics within each center are required to achieve homogeneity, which can be solved by increasing the sample size to ensure reliability of the final pooled samples. The follow-up rates in clinical studies are another key issue affecting reliability of the study results. Specialized staff in each center were assigned the task of following included cases, establishing case study databases, and recording various contact methods to guarantee a follow-up rate of over 80%.

The incidence of DDH in China is 1.52% (26).The large basis of the annual newborn population and the large number of patient samples in China are conducive to effectively accumulating study samples within a short period of time, which increases the feasibility of this study protocol. Our research question stems from novel ideas in clinical practice, and this study is the first to suggest that the inverted limbus cannot be absorbed after closed reduction for DDH and is a risk factor of RAD. This study is also the first to discuss the effects of RLI based on clinical practice. By conducting this study, we aim to clarify the effect of RLI on acetabular development after closed reduction of DDH, define the timing and indications for surgical interventions of RLI after closed reduction of DDH, and to inform revision of the criteria for an acceptable closed reduction in DDH treatment so that the incidence of OA caused by RAD after DDH treatment can be reduced in clinical practice.

The exact mechanism by which RLI causes RAD after closed reduction for DDH remains unclear. Wang et al. (27) used latex pads to simulate the inverted limbus embedded between the femoral head and acetabulum of the model hip joint and performed mechanical experiments to confirm that the local contact pressure at the location of the latex pads was significantly increased. Hence, we speculated that persistent imbalance in pressure between the acetabulum and femoral head caused by RLI after closed reduction for DDH is a major factor contributing to RAD. In future studies, we will further perform biomechanical experiments at the cellular level using animal models to provide insights into the mechanism of action of RAD in limbus inversion. It is of great clinical significance to reveal the molecular mechanism by which RLI causes acetabular dysplasia and to obtain robust evidence to guide clinical decisions.

## 4. Ethical considerations

### 4.1. Research ethics approval

The registration of clinical trials was completed prior to this study protocol (registration number: ChiCTR1900020996) and was approved by the Medical Ethics Committee of Shengjing Hospital of China Medical University.

This study will recruit pediatric patients who have undergone closed reduction. Pediatric patients who meet al.l inclusion criteria and do not meet any exclusion criteria will be considered for this study. Prior to enrollment in the study, researchers will explain the objectives and content of the study in detail to the guardians of study participants as well as discuss the surgical risks and benefits of RLI clearance after closed reduction; researchers will ensure that the guardians are fully informed of the study. The guardians of the study participants must provide written informed consent before any research activity may begin. Participation in this study is voluntary and may be withdrawn by the participants' guardians at any time.

### 4.2. Data collection and management

A medical record registration system has been developed according to the study protocol and data analysis plan. To ensure the integrity of critical data, mandatory fields are pre-specified. Instructions for completing medical record registration forms may be accessed within the system, and quality assurance protocols have also been developed. Designated, well-trained qualified investigators will ensure accurate recording of data for all included patients in the medical record registration system. The investigator will review and submit data to specialized data administrator personnel for data entry and management. To ensure the accuracy of the data, double data entry will be performed by two separate investigators; entered data will be verified and validated by software which will prompt for manual error corrections. After the central database administrator confirms that the database is accurate and error-free, the database will be locked. The locked data files are then submitted to an independent statistical team for analysis.

### 4.3. Confidentiality

All study-related information will be securely stored at the study site in areas with access restricted to the research team. All records containing names or other personally identifying information, such as locator forms or informed consent forms, will be stored separately from study records identified by study subject ID numbers. All local databases must be secured using password-protected access systems.
